# Enhanced biodegradation of phenol in a novel cyclic activated sludge integrated with a rotating bed bioreactor in anoxic and peroxidase-mediated conditions

**DOI:** 10.1039/c7ra12997a

**Published:** 2018-02-07

**Authors:** Mojtaba Pourakbar, Gholamreza Moussavi, Kamyar Yaghmaeian

**Affiliations:** Department of Environmental Health Engineering, Faculty of Medical Sciences, Tarbiat Modares University Tehran Iran ppourakbar@yahoo.com moussavi@modares.ac.ir +98 21 82883863 +98 21 82883827; Department of Environmental Health Engineering, School of Public Health, Tehran University of Medical Sciences Tehran Iran kyaghmaeian@tums.ac.ir; Center for Water Quality Research, Institute for Environmental Resarch, Tehran University of Medical Sciences Tehran Iran

## Abstract

Cyclic activated sludge integrated with a rotating bed bioreactor (CASIR) was used for phenol biodegradation. The effects of phenol loading rate, mixed liquor suspended solids (MLSS) concentration, media filling ratio, hydraulic retention time (HRT) and salinity were investigated for phenol degradation and COD removal. In the second phase of the study, the microbial content of the bioreactor was induced by hydrogen peroxide injection for *in situ* generation of peroxidase. For investigating the above-mentioned parameters, the bioreactor was operated for 535 days and residual phenol, nitrate and COD were measured daily. The variation of the dehydrogenase activity and peroxidase activity of suspended biomass and attached film were also monitored during the bioreactor operation. Complete degradation of phenol at the loading rate of 667 g m^−3^ d^−1^ was achieved in anoxic conditions. Addition of media to the bioreactor to form active attached biofilm led to the increase in tolerance of the bioreactor on organic loading shocks. It was found that increasing the salinity of the wastewater did not affect the performance of the bioreactor. Investigating dehydrogenase activity proved that the attached biofilm was more involved in phenol degradation, compared with the suspended biomass. However, after switching to peroxidase-mediated conditions, the organic loading tolerance of the bioreactor considerably increased and complete degradation of phenol at the loading rate of 2000 g m^−3^ d^−1^ was reached. After adaptation of the microorganisms for hydrogen peroxide, the peroxidase activity of 290 U g_biomass_^−1^ was observed in the bioreactor. Accordingly, the H_2_O_2_-induced microbial cells in cyclic activated sludge could be considered as a promising technique for enzymatic degradation of phenol and corresponding COD.

## Abstract 2

Cyclic activated sludge integrated with a rotating bed bioreactor (CASIR) was used for phenol biodegradation.

## Graphical abstract

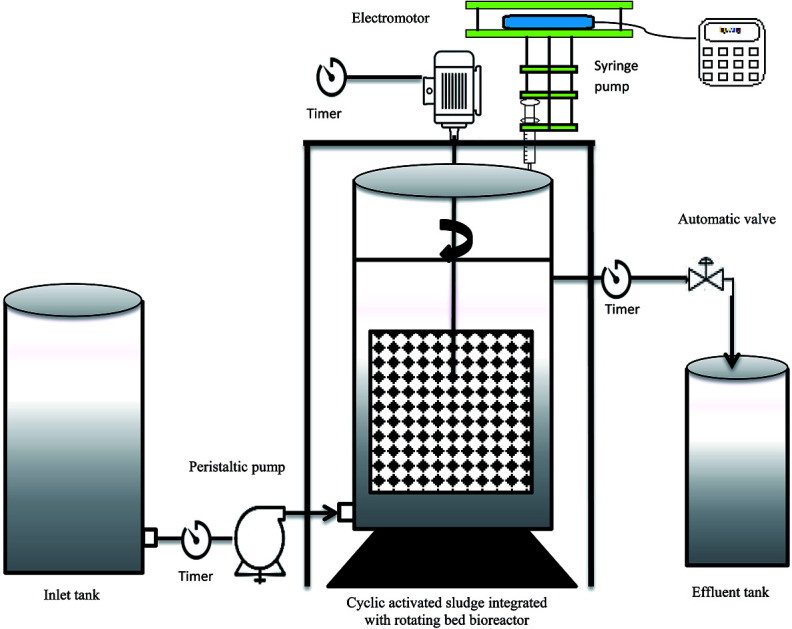

## 1. Introduction

Phenol is regarded as a toxic aromatic compound that has been placed on the priority pollutant list by the United States Environmental Protection Agency (USEPA). Phenolic compounds are commonly detected in industrial wastewaters from industries such as petrochemical, pharmaceutical, textile, and paint, and are threatening to the environment. It is reported that almost 700 million tons of phenol are produced annually.^1^ Excessive discharge of phenol through wastewater into aqueous ecosystems causes serious environmental pollution, even at low concentrations.^2^ The EPA has regulated the concentration of phenol in wastewater below 2 mg L^−1^ in order to protect human health from the potential toxic effects caused by exposure to phenol.^3^ In general, it is of vital importance to remove phenol from wastewater prior to discharge to the environment. However, phenol is not easily removed from its dissolved state in water due to its considerable molecular polarity, which brings about its high solubility over 10 wt%. To protect human health and the ecosystem, the development of an efficient and environmentally-friendly technique to remove phenol from its aqueous solution is of great importance.

Several investigations have been carried out to remove phenol from industrial phenol-laden wastewaters, including a combination of physical, chemical and biological processes. Conventional physicochemical technologies for phenol removal include oxidation, ion-exchange, adsorption, electrocoagulation, *etc.*^4–6^ On the other hand, biological processes have been preferred to physical and chemical technologies due to their ability to destroy a wide range of pollutants in an environmentally friendly and cost effective way.^7–9^ Several studies have been conducted regarding the biodegradation of phenol from wastewater.^10–13^ Although phenol degradation can occur in both aerobic and anaerobic conditions, studies have shown that phenol degradation in aerobic conditions is better than in anaerobic conditions because phenol can inhibit the anaerobic biological processes.^14^ However, due to the need for aeration of the wastewater, aerobic processes have high energy consumption. On the other hand, phenolic compounds can be found together with other pollutants such as nitrates in wastewater. The use of nitrate as the final electron acceptor in the oxidation of phenol, as the carbon source of microorganisms, makes the biological process more interesting.^15^ Therefore, anoxic degradation of phenol from wastewater leads to the removal of both phenol and nitrate, and the problems associated with the aeration of the wastewater are eliminated.

Microorganisms, either aerobic or anoxic, in phenol biological oxidation can be attached to a medium or suspended in the bioreactor. Several studies have been conducted using suspended and attached growth microorganisms for phenol degradation.^13,16–18^ It has been reported that attached biofilm is more resistant to toxic compounds compared to suspended biomass; for example, Tziotzios *et al.*^19^ reported that a packed bed reactor led to higher phenol degradation, as compared with a suspended growth bioreactor, especially when the packed bed reactor is operated in fill and draw mode. Yusoff *et al.*^20^ reported in 2016 that in hybrid growth systems (including suspended and attached growth systems) the inhibitory effect of phenol towards the biochemical activities of the microorganisms was minimized.

Cyclic operation is a modification of the conventional activated sludge process, which is conducted in a variable volume reactor. The process operates with sludge in a single reactor basin to accomplish both biological treatment and solid–liquid separation. The sequencing batch reactor (SBR) is the commonly used cyclic activated sludge process. The sequencing continuous reactor (SCR) was subsequently developed as the modification of the SBR. Considering the advantages of attached biofilm, such as larger microbial diversity and biochemical activities, having large biomass contained per volume of bioreactor, higher degradation rate, higher resistance to toxic shocks and prevention of biomass washout,^21^ a hybrid suspended biomass and attached biofilm bioreactor were developed to degrade phenol from wastewater. Cyclic Activated Sludge Integrated with a Rotating bed (CASIR) is a newly developed and modified bioreactor of SCR having both attached growth and suspended growth.

In recent years, enzymatic biodegradation of recalcitrant compounds have been conducted by several researchers. Enzymatic biodegradation is considered as a promising approach for pollutant biodegradation due to substrate specificity, efficiency and the ease of handling.^22^ In enzymatic biodegradation, microorganisms generate intracellular and extracellular enzymes.^23^ The presence of peroxidase enzyme, an oxidoreductase enzyme, in the wastewater could lead to the generation of free radicals initiating bond-cleavage reactions and subsequent degradation of the organic compounds would occur.^23^ Bacterial biostimulation for the generation of peroxidase enzyme has been reported to be effective for pollutant degradation. Bacterial biostimulation by hydrogen peroxide causes peroxidase generation to protect the living cells.^24^ Therefore, addition of H_2_O_2_ to the bioreactors is an interesting choice for *in situ* generation of peroxidase.

Accordingly, the present study is aimed toward investigating the performance of the CASIR bioreactor for phenol degradation in anoxic conditions and compares the performance with peroxidase-mediated conditions. The effect of inlet phenol concentration, Mixed Liquor Suspended Solids (MLSS), media filling ratio, Hydraulic Retention Time (HRT), and salinity were evaluated on phenol and COD removal.

## 2. Experimental

### 2.1. Synthetic wastewater

The synthetic wastewater was generated by dissolving a known amount of phenol as the sole carbon source in dechlorinated tap water. The required nutrients for microbial metabolism were added into the synthetic wastewater to maintain the COD/N/P ratio of 100/5/1. The stock nutrient solution consisted of 15 g KH_2_PO_4_, 5 g K_2_HPO_4_, 120 g NH_4_Cl, 10 g CaCO_3_, 12 g (NH_4_)_2_HPO_4_, and 10 g NaHCO_3_ in one liter of tap water. The pH of the synthetic wastewater was maintained at neutral conditions. All the chemicals used in the present study were of analytical grade and were purchased from Merck.

### 2.2. Experimental setup

**Fig. 1:**
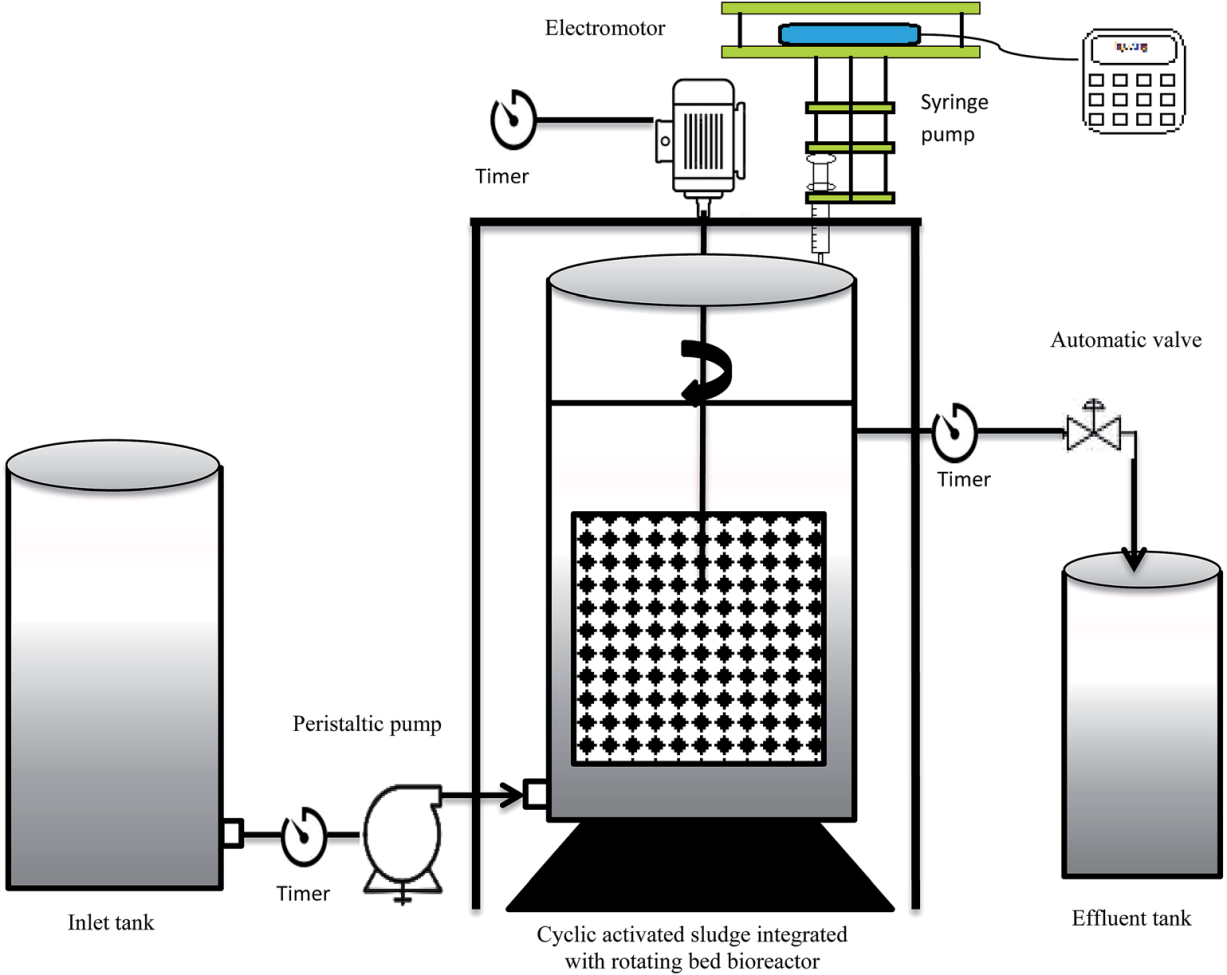
Schematic of the cyclic activated sludge integrated with rotating bed bioreactor.

The experiments were carried out in a cylindrical glass with a diameter of 20 cm and height of 36 cm (25 cm fixed height). Fig. 1 illustrates the schematic of the CASIR bioreactor. The feed into the bioreactor was conducted by a peristaltic pump (WATSON MARLOW 101U/R). A perforated basket was used to hold polyurethane foam (PUF) cubes (1 cm^3^) with density of 35 kg m^−3^ and specific surface area of 600 m^2^ m^−3^. The media package was rotated inside the bioreactor with an electromotor (14 W), which was connected by a steel shaft. Supernatant decantation was conducted using a solenoid valve controlled by a timer. Another timer was also connected to the electromotor to control the reaction and settling times in the bioreactor. The bioreactor was operated in cyclic mode and each cycle lasted for 3 h, consisting of reaction (2 h), settling (0.75 h), and decanting (0.25 h). Injection of synthetic wastewater was carried out continuously from the bottom of the bioreactor.

### 2.3. Inoculum, start-up and operation

The bioreactor was filled with activated sludge used for catechol degradation in our previous study.^25^ The CASIR bioreactor contained a mixed microbial culture with both attached growth and suspended growth. The concentration of MLSS was regulated at 4000 ± 200 mg L^−1^. Both the suspended biomass and attached thin active biofilm were responsible for phenol degradation in the CASIR bioreactor. Suspension of the biomass in the reaction step was obtained by rotation of the media package. To determine the amount of attached biofilm, 5 pieces of media were withdrawn from the middle of the basket and the attached biofilm was washed with 200 mL of nutrient solution to detach the biofilm. The concentration of the nutrient solution containing the detached biofilm was measured and the amount of biofilm attached to each media was calculated to be 58.08 mg. Considering the total of 125 pieces of media in the bioreactor, the total attached biofilm was calculated to be 7.2 g. Further measurement of the biofilm during different operational times revealed that there was not a considerable difference in the amount of attached biofilm.

In the present study, nitrate was used as the final electron acceptor, and the inlet nitrate concentration was determined based on the inlet COD concentration and the COD/nitrate ratio was selected to be 1.^25,26^ Initially, the reactor was operated with 100 mg L^−1^ phenol. The start-up phase was assumed to be complete after reaching 100% phenol removal. After the start-up period, the effects of inlet phenol concentration (100–800 mg L^−1^), MLSS (4–7 g L^−1^), media filling ratio (0–40%), HRT (12–24 h), and salinity (1–20 g L^−1^) were investigated.

In the second phase of the study, the bioreactor was switched from reducing conditions (anoxic) to oxidizing conditions in which hydrogen peroxide acted as the final electron acceptor. To get these conditions, the nitrate concentration in the effluent wastewater was reduced gradually and hydrogen peroxide was added using a syringe pump (Fig. 1).

Table 1 summarizes the experimental phases. Media filling ratio is defined as the volume of the media to total working volume of the bioreactor. Each experimental condition was set up to reach pseudo-steady state conditions in terms of effluent COD. Pseudo-steady state conditions were assumed to be reached when the effluent COD fluctuation was less than 3% in at least 3 consecutive days of operation (3HRT). All the experiments were conducted at room temperature (20 ± 2 °C).

**Table 1:** Experimental and operation conditions

Salinity (g L^−1^)	Media filling ratio (%)	HRT (h)	MLSS (g L^−1^)	Inlet phenol concentration (mg L^−1^)	Operation condition	Day
Tap water	40	24	4	100	Start-up	1–21
Tap water	40	24	4	100–800	Inlet phenol concentration	22–94
Tap water	40	24	4–7	800	MLSS	119–183
Tap water	0–40	24	4	500	Media filling ratio	183–300
Tap water	40	12–24	4	500	HRT	301–354
0–20	40	24	4	800	Salinity	355–409
Tap water	40	12	4	500	H_2_O_2_ injection	410–510

### 2.4. Analytical methods

The samples were collected daily and were analyzed for residual phenol concentration, COD, nitrate and nitrite. The MLSS of the bioreactor was also monitored on a routine basis. The dehydrogenase activities (DHA) of both suspended biomass and attached biofilm were also routinely monitored. The decanted supernatant was filtered to remove particles with a 0.45 μm pore size filter prior to measuring phenol, COD, nitrate and nitrite concentrations. The phenol concentration was measured spectrophotometrically using the colorimetric 4-aminoantipyrine procedure as given in the standard methods^27^ using a Unico-UV 2100 UV/vis spectrophotometer. pH was measured using a Jenway 3505 pH meter. All the other parameters including COD, nitrate, nitrite, and the MLSS were measured according to the procedures given in standard methods.^27^

The performance of the CASIR bioreactor was measured based on the COD and phenol removal efficiencies as a function of inlet phenol and COD concentrations (eqn (1)). In addition, the phenol loading rate was calculated using the following equations:1
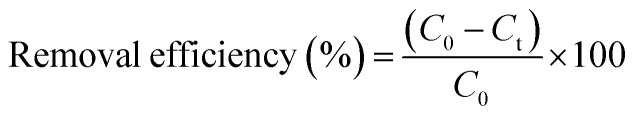
2
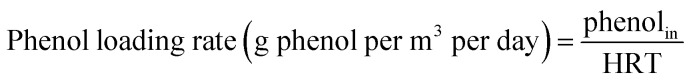
where, *C*_0_ and *C*_t_ are the phenol and COD concentrations in the inlet and outlet of the bioreactor, respectively.

The dehydrogenase enzyme activity (DHA) of the suspended biomass and attached growth was measured at the end of each experimental run when the reactor was in steady-state conditions.

Triphenyl tetrazolium chloride (TTC) was used as the hydrogen acceptor for the dehydrogenase test.^28^ TTC in the presence of dehydrogenase enzymes produces a red color, which is due to the generation of 1,3,5-triphenyltetrazolium formazan (TF). This red color could be measured spectrophotometrically at 492 nm. The generated TF is water insoluble and it was extracted using toluene.^29^ TF was used to establish a standard curve for absorbance *vs.* TF concentration. Tris–HCl (pH = 7.6) was also used to control the pH of the samples. Mixed liquor samples taken from the bioreactor were added to tubes containing 2 mL of tris–HCl buffer, 2 mL of glucose solution (0.1 M), and 2 mL of TTC (5%). The tubes were then incubated in a water bath at 37 °C and incubated for 24 h. After the incubation period, the reaction was stopped by adding 2 mL of sodium dithionate (Na_2_S_2_O_4_). Then, 5 mL of toluene was added to the tubes and the contents of the tubes were centrifuged at 5000 rpm for 10 min afterward, the supernatant was used for TF measurement. Finally, DHA was expressed in terms of μg TF per g_biomass_ per day.

Peroxidase activity (PA) was measured based on the procedure given by Tandjaoui *et al.*,^30^ with some modifications. Based on this method, 10 mL of the mixed liquor was taken and centrifuged at 10 000 rpm at 4 °C for 20 min. Afterward, 40 μL of the supernatant was taken and 350 μL buffer phosphate (0.3 M, pH = 7), 100 μL guaiacol and 160 μL H_2_O_2_ solutions were added in a UV-Vis spectrophotometer. Then, the changes in the absorbance at 470 nm were monitored for 5 min. The PA was expressed as U g_biomass_^−1^ indicating the amount of peroxidase produced per unit of biomass, which oxidized 1 μmol of substrate per min at 25 °C.^30^

## 3. Results and discussion

### 3.1. Bioreactor start-up and the effect of inlet phenol concentration

**Fig. 2:**
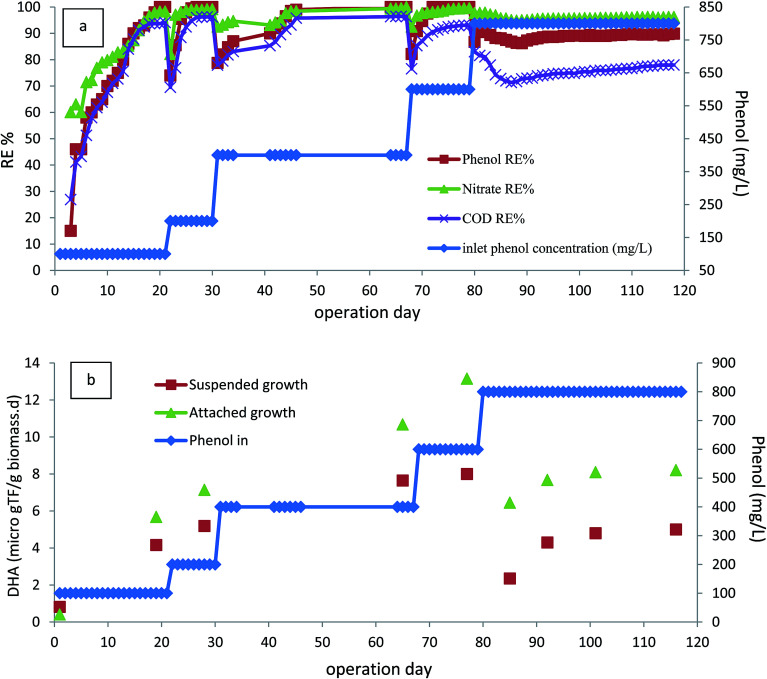
(a) Start-up and the effect of inlet phenol concentration on phenol, COD and nitrate removal. (b) Variation of DHA within the start-up stage and varying inlet phenol concentration. (HRT = 24 h, media filling ratio = 40%, MLSS = 4000 mg L^−1^).

The performance of the bioreactor was investigated for inlet phenol concentrations of 100–800 mg L^−1^, corresponding to COD concentrations of 230–1900 (mg L^−1^) at an HRT value of 24 h. Fig. 2a shows the phenol and corresponding COD removal efficiencies within 120 days of operation. The bioreactor was started up by injecting with inlet phenol concentration of 100 mg L^−1^. As shown, the removal efficiency of phenol after 15 days of operation reached 90%, and continuing the operation of the bioreactor led to the complete removal of phenol after 20 days. In addition, COD removal efficiency of almost 94% was reached after this period of operation. Furthermore, nitrate was also reduced and almost 98% of nitrate removal efficiency was reached. After reaching steady-state conditions within this time, the concentration of inlet phenol increased to 200 mg L^−1^. As shown in Fig. 2a, there was a small reduction in the removal efficiency in the first few days, but this was very short-lived and the efficiency improved to complete removal after 5 days of operation, showing the completion of the start-up phase. The rapid time of start-up could be due to inoculating the bioreactor with adapted phenolic compound degrader biomass. After the start-up section, the bioreactor was operated at inlet phenol concentrations of 400, 600, and 800 mg L^−1^. Initial phenol concentration is of great importance in the performance of the continuous biodegradation process, since some hydrocarbon contaminants such as phenol are known to have inhibitory effects on the activity of the biomass. At the inlet phenol concentration of 600 mg L^−1^, complete degradation of phenol along with 93% COD removal was reached, but increasing the inlet phenol concentration to 800 mg L^−1^ with a corresponding inlet COD concentration of 1900 mg L^−1^ led to decreased phenol and COD removal efficiency below 90% and 74%, respectively, after 15 days of operation. This implies that the critical phenol concentration for the anoxic bioreactor was 800 mg L^−1^ at HRT value of 24 h (800 g m^−3^ d^−1^). Concentrations larger than this have an inhibitory effect on the activity of the biomass and may lead to the accumulation of intermediates in the bioreactor.^31^ Nitrites in the effluent were also measured during this phase, and no nitrite accumulation was observed at inlet phenol concentrations up to 600 mg L^−1^. However, increasing the inlet phenol concentration to 800 mg L^−1^ led to the appearance of 0.8 mg L^−1^ nitrite in the effluent.

As shown in Fig. 2a, the COD removal shows the same trend as phenol removal, implying that phenol is metabolized for energy production *via* active biomass and has been completely biodegraded.

Sarfaraz *et al.*^32^ reported that complete degradation of 649 mg L^−1^ phenol was reached in an anoxic granular SBR with 12 h cycle length. Bajaj *et al.*^33^ investigated the performance of an anoxic suspension bioreactor for phenol degradation and found that 207 mg L^−1^ phenol was almost completely degraded; when the inlet phenol concentration increased to 329 mg L^−1^ there was significant reduction in both phenol and COD removal. Rosenkranz *et al.*^34^ investigated an anaerobic SBR for degradation of phenol in synthetic wastewater; they reported that 800 mg L^−1^ of phenol was almost completely degraded at reaction time of 50 h. Liu *et al.*^13^ reported that 300 mg L^−1^ phenol was 99% biodegraded in an anoxic–aerobic bioreactor; most of the phenol was degraded in the anoxic stage. As seen in the literature, the performance of the anoxic CASIR is better compared to other similar anoxic bioreactors. This could be due the use of an acclimated culture of active microorganisms in the bioreactor and the presence of active attached biofilm, which had a considerable effect on the overall performance of the bioreactor. To prove this fact, DHA of the suspended biomass and attached biofilm was measured at different operational runs with varying inlet phenol and COD concentrations. Fig. 2b illustrates the DHA of the CASIR process within 118 days of operation. The lowest DHA in the bioreactor was at the start-up stage; after completion of the start-up stage and increasing the inlet phenol concentration, the DHA of suspended biomass was also increased from 0.82 μg TF per g_biomass_ per day in the first day of operation to 8 μg TF per g_biomass_ per day on day 79 (inlet phenol concentration of 600 mg L^−1^). The same trend was observed for attached biofilm, but the DHA values at the first day and 79^th^ day were 0.41 and 13.15 μg TF per g_biomass_ per day, respectively. This increase in DHA shows that the microorganisms consume the substrate for their metabolic activities. This may be due the fact that the phenol concentration up to the given point can stimulate the DHA and cause enhanced bacterial growth and biodegradation rate.^35,36^ When the inlet phenol concentration increased to the critical point of 800 mg L^−1^, there was a significant reduction in DHA, indicating that the bioreactor is operating at the tolerance threshold. The reduction in phenol and COD removal efficiencies at the higher loading rates implies that the microorganisms are acting at their maximum capacity in the metabolic activities.^37^ In our previous study, the SCR bioreactor was used for petroleum hydrocarbon degradation, DHA activity was also reduced at critical inlet concentration.^38^

### 3.2. The effect of the concentration of mixed liquor suspended solids

**Fig. 3:**
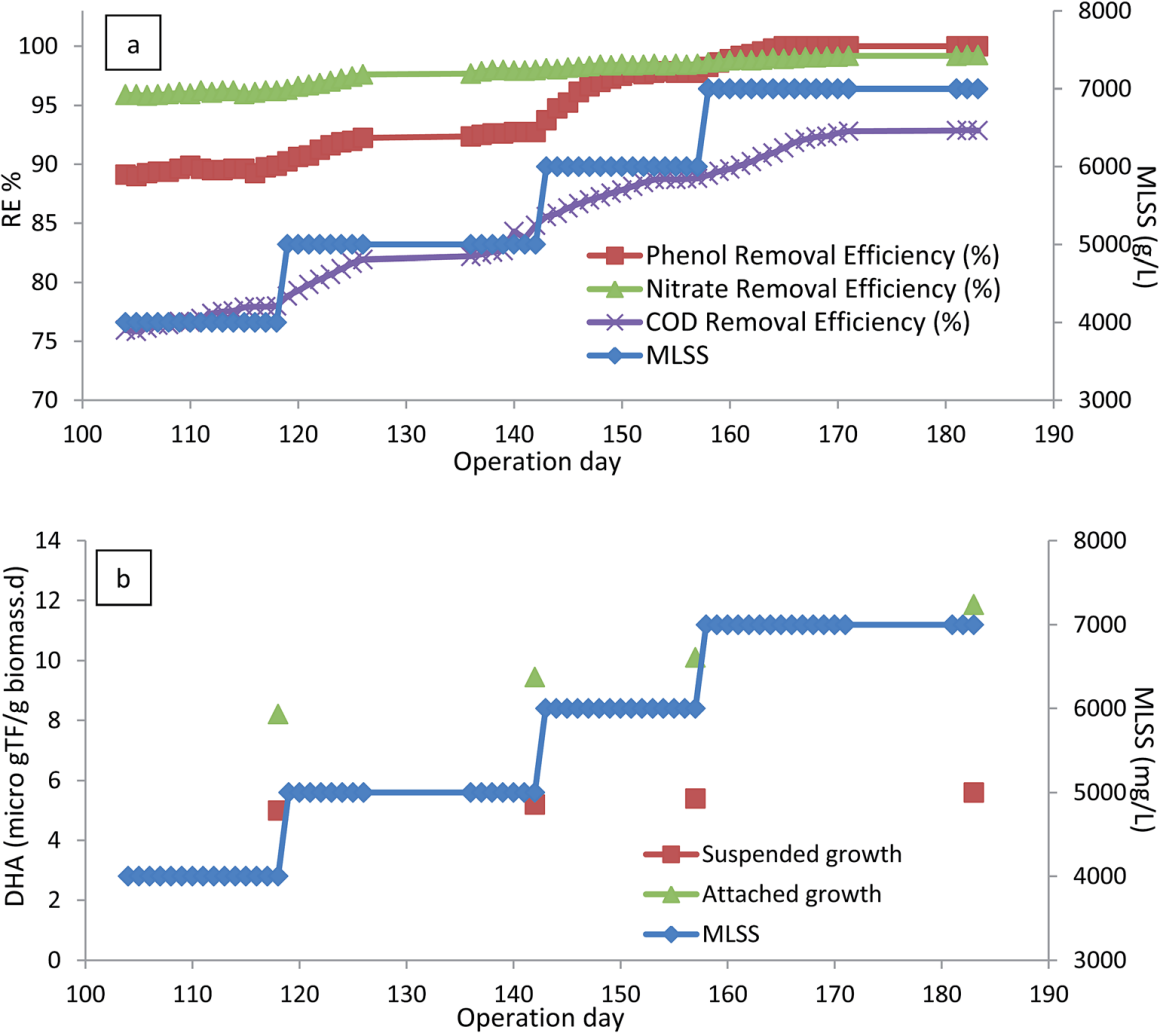
(a) Effect of MLSS on the performance of the bioreactor. (b) Variation of DHA as a function of MLSS. (HRT = 24 h, media filling ratio = 40%, inlet phenol concentration = 800 mg L^−1^).

The effect of MLSS concentrations of 4–7 g L^−1^ was investigated in the following conditions: inlet phenol concentration = 800 mg L^−1^, HRT = 24 h, and media filling ratio = 40%. When the MLSS of the bioreactor was increased to 5 g L^−1^, there was a slight increase in the removal efficiencies of phenol, COD, and nitrate. Increasing the MLSS concentration up to 7 g L^−1^ leads to the increase in phenol removal efficiency. As shown in Fig. 3a, 800 mg L^−1^ phenol was completely biodegraded and 92% COD removal was reached at this stage. The reason for higher phenol and COD removal at higher MLSS is due to the higher number of active microorganisms present in the bioreactor.^39^ By increasing the concentration of microorganisms in the bioreactor, the ratio of inlet food to microorganisms became smaller, causing an increment in the COD and phenol removal efficiencies.^40^ Although there were higher removal efficiencies for phenol and COD at higher concentrations of suspended biomass, bioreactor operation at higher levels of suspended biomass was difficult, and clogging of the outlet due to the sludge rising was a common problem.

Fig. 3b illustrates the performance of the CASIR bioreactor with DHA as a function of MLSS concentration. As shown in the figure, there was a slight increase in DHA of the suspended biomass and attached biofilm. This is due to the fact that further degradation of phenol and its intermediates reduces the toxicity of phenol to the microorganisms and therefore stimulates DHA. In addition, the increase in the DHA activity of the attached biofilm is greater than that of the suspended biomass. This is due to the fact that when calculating the DHA of the suspended biomass, the value of mg_biomass_ in the denominator increases and therefore the overall DHA is reduced. On the other hand, DHA of the biofilm increases, which is due to the reduction in the phenol concentration present in the reactor. Immobilized biomass on an inert surface allows more active working organisms in the bioreactor with more stable operation; this is achieved by improving the retention of microorganisms, allowing the reactor to cope with a greater concentration of biomass. Therefore, the integration of attached growth with suspended growth in the CASIR bioreactor makes the process more stable and effective in phenol degradation.

### 3.3. The effect of media filling ratio

**Fig. 4:**
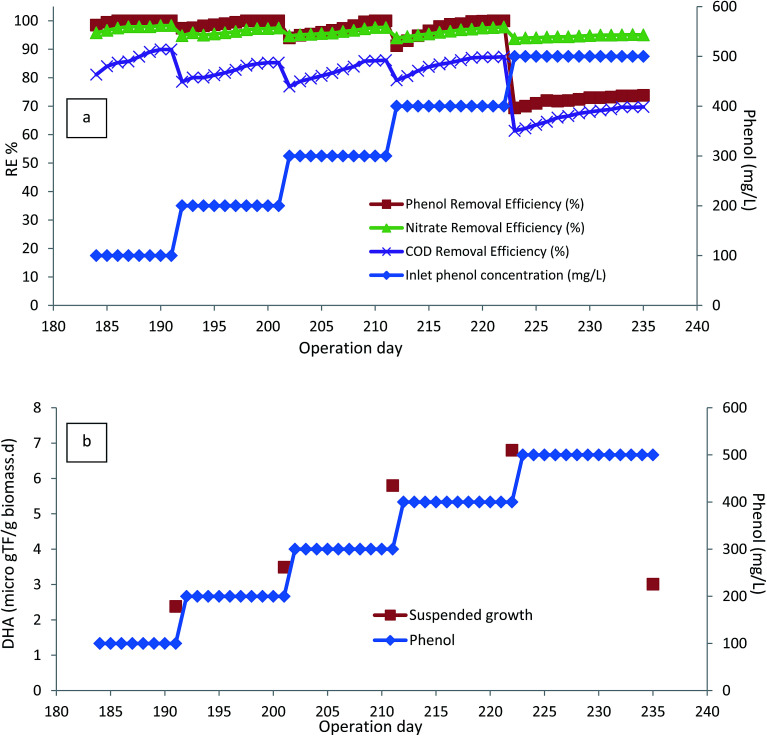
(a) Performance of the bioreactor without the presence of media. (b) DHA of the bioreactor without the presence of media. (HRT = 24 h, media filling ratio = 0%, MLSS = 4000 mg L^−1^).

In order to investigate the effect of the presence of attached biofilm on the performance of the bioreactor, all the media were taken out and the inlet phenol concentration was reduced to 100 mg L^−1^. Fig. 4a illustrates the performance of the bioreactor without any attached biofilm growth at inlet phenol concentrations of 100–500 mg L^−1^. As shown in the figure, the process could completely degrade inlet phenol concentration of 400 mg L^−1^, and almost 87% COD removal was achieved, but further increase of inlet phenol concentration to 500 mg L^−1^ led to the reduction of phenol to 73.8% and COD removal to 69.74%. DHA was also decreased from 6.8 to 3.01 μg TF per g_biomass_ per day (Fig. 4b).

In the next section, in order to see the effect of the presence of biofilm in the bioreactor, the media filling ratio increased to 10%. The presence of media with attached growth biofilm, increased the removal efficiency of phenol to 95.8% after 15 days of operation, and COD removal increased to 88.06%. Further increase of the media filling ratio to 20% led to 100% removal of phenol and 91% of COD was also removed. After reaching steady-state conditions, the media filling ratio increased to 30 and 40% and finally 94.28% of COD was removed. It can be seen that phenol, nitrate and COD removal efficiencies were increasing just after inserting the media, and steady-state conditions were quickly reached after increasing the media filling ratio.

**Fig. 5:**
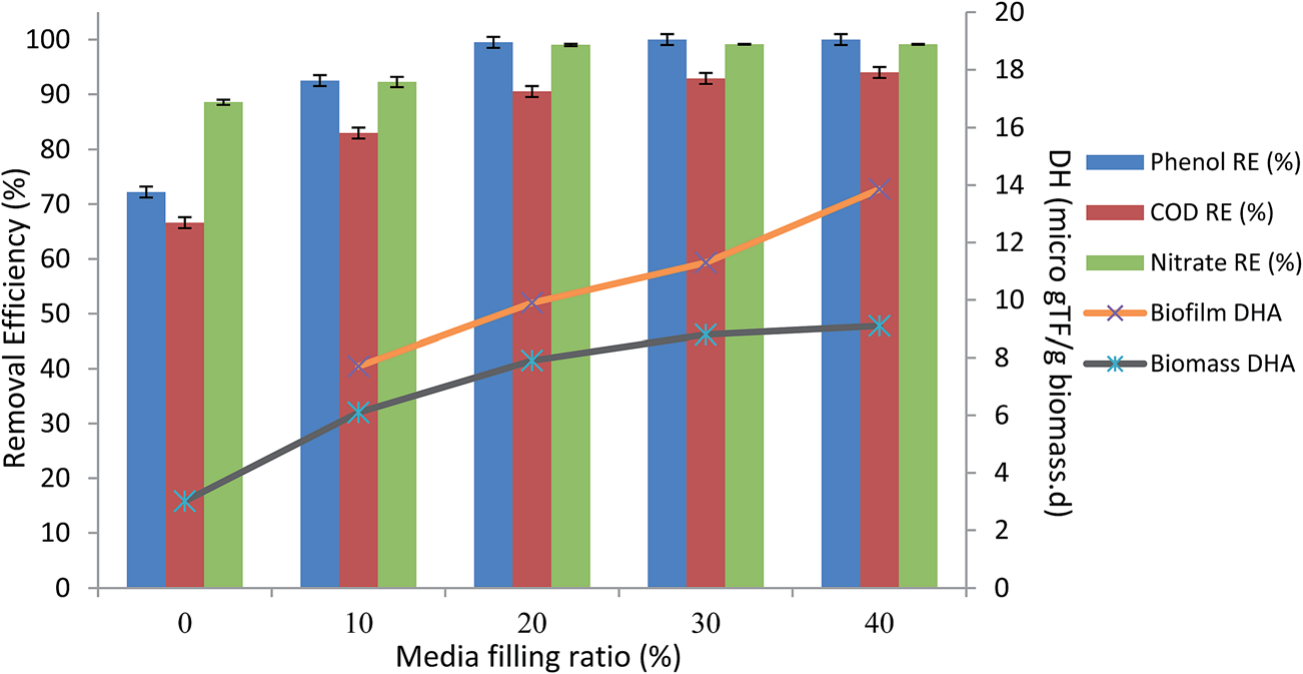
Average steady-state phenol, COD, nitrate reduction and DHA variation in various media filling ratios. (HRT = 24 h, inlet phenol concentration = 500 mg L^−1^, MLSS = 4000 mg L^−1^).

Fig. 5 illustrates the average steady-state phenol, COD, and nitrate removals at varying media filling ratios. As shown in the figure, 100% removal of 500 mg L^−1^ phenol was reached by 30% media filling ratio. More mineralization of organic compounds in the bioreactor occurred by further increasing the media filling ratio. Biofilm generation in the bioreactor caused the increment in DHA, as seen in Fig. 5, due to the presence of more active working microorganisms, which caused a reduction in the toxicity of the phenol.^16^

Integration of suspended biomass with attached biofilm in the CASIR bioreactor also led to improvement of the quality of the effluent in the case of Total Suspended Solid (TSS). TSS of the effluent when there was no media in the bioreactor was measured to be 46 mg L^−1^ (HRT = 24 h, media filling ratio = 0%, inlet phenol concentration = 500 mg L^−1^, and MLSS = 4000 mg L^−1^), but addition of the media and formation of the biofilm improved the performance of the bioreactor and the effluent TSS was reduced to 6.5 mg L^−1^. This is in accordance with the findings of other researchers.^17^ Highly efficient removal of TSS indicates that the integration of biofilm and suspended biomass was highly effective as an adsorbent, filtration unit and biofilm attachment mechanism such that the system was able to accept a higher organic load in the influent.

### 3.4. The effect hydraulic retention time

Providing sufficient contact time between microorganisms and contaminants is one of the important parameters in biological wastewater treatment. Therefore, the effects of HRT values of 24 h (corresponding to the organic loading rate of 500 g m^−3^ d^−1^), 18 h (corresponding to the organic loading rate of 667 g m^−3^ d^−1^), and 12 h (corresponding to the organic loading rate of 1000 g m^−3^ d^−1^) on the performance of the CASIR bioreactor were investigated. This parameter was investigated at inlet phenol concentration of 500 mg L^−1^, media filling ratio of 40%, and MLSS of 4000 mg L^−1^. To better understand the effect of HRT, the average steady-state phenol, COD and nitrate removal efficiencies along with corresponding DHA are presented in Table 2, which shows that reducing the HRT to 18 h, does not affect the removal efficiency of phenol, but there is a slight reduction in COD and nitrate removal efficiencies. DHA of the biomass and biofilm also increased in the bioreactor; however, further decrease in HRT contributed to the reduction of phenol, COD and nitrate removal efficiencies. Marañón *et al.*^41^ investigated the treatment of coke wastewater containing phenol in a SBR system and observed a removal efficiency of 99% for a phenol concentration of 207 mg L^−1^ and an HRT of 115 h, corresponding to a phenol loading rate of around 1.67 g m^−3^ d^−1^; for greater concentrations, they found that phenol significantly inhibited COD removal. There are also some studies reporting higher phenol loading rates, for instance, Uygur and Kargi^42^ reported more than 95% COD removal in a four-step SBR (anaerobic/oxic/anoxic/oxic) at phenol concentrations up to 400 mg L^−1^ (loading rate of 1600 g m^−3^ d^−1^). Moussavi *et al.*^43^ investigated the aerobic moving-bed sequencing batch reactor for phenol degradation; it was reported that almost 94% of phenol was degraded at the loading rate of 83.4 g m^−3^ h^−1^. However, the anoxic degradation of phenol with high organic loading rate is rare. Therefore, the CASIR bioreactor could be considered a promising process for the simultaneous removal of phenol and high concentrations of nitrate.

**Table 2:** Average steady-state phenol, COD and nitrate removal efficiencies as a function of HRT (inlet phenol concentration = 500 mg L^−1^, MLSS = 4000 mg L^−1^)

Attached biofilm DHA (μg TF per g_biomass_ per day)	Suspended biomass DHA (μg TF per g_biomass_ per day)	Nitrate RE ± SD (%)	COD RE ± SD (%)	Phenol RE ± SD (%)	Phenol loading rate (g m^−3^ d^−1^)	HRT (h)
13.86	9.1	95.13 ± 0.07	93.99 ± 0.36	100	500	24
14.68	9.5	91.69 ± 2.39	89.87 ± 4.3	100	667	18
6.9	4.1	63.75 ± 0.57	49.73 ± 7.62	59.52 ± 9.6	1000	12

### 3.5. The effect of salinity

**Fig. 6:**
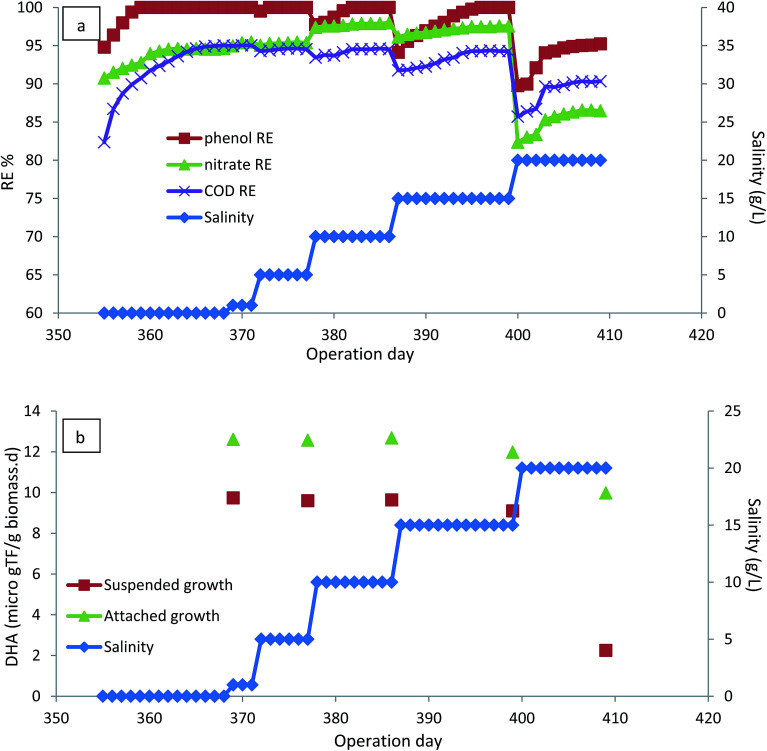
(a) The effect of salinity on the performance of the CASIR bioreactor. (b) The effect of salinity on the DHA of biomass and biofilm. (HRT = 24 h, inlet phenol concentration = 500 mg L^−1^, MLSS = 4000 mg L^−1^).

Salts are important raw materials in the chemical industries, various manufacturing processes and other industrial operations. Consequently, the presence of salts in industrial wastewater is inevitable.^44^ The occurrence of phenolic compounds in saline wastewater may limit the microbial degradation of phenol and make conventional biodegradation processes ineffective due to the adverse effect of salts on microbial metabolism;^16,45^ therefore, the effect of the presence of salinity (1–20 g L^−1^) on the performance of the CASIR bioreactor was investigated. To investigate the effect of salinity, the HRT of the bioreactor was increased to 24 h, with inlet phenol concentration of 500 mg L^−1^ (organic loading rate of 500 g m^−3^ d^−1^) and the operation continued until steady-state conditions were reached. As seen in Fig. 6a, the bioreactor could recover and re-attain performance after having an organic shock load of 1000 g m^−3^ d^−1^ (HRT of 12 h). The bioreactor could preserve its performance after the organic load was applied, revealing the high capacity of the developed reactor to amortize the shock load received. Quick recovery of the bioreactor from the organic shock load can be related to the presence of a high-density, active microbial population in the reactor, and the use of media that improves mass transfer and thus biodegradation. In addition, another reason for the observed stability of the CASIR bioreactor under organic shock loads was the batch mode of effuent decantation as well as high concentration of attached biomass, which is able to tolerate higher organic loads.^46,47^

After recovery of the bioreactor and re-attaining the steady-state conditions, the salinity of the inlet wastewater increased. For all the examined salinity levels, the increase of salinity led to the reduction of removal efficiencies, but the bioreactor again reached the steady-state and 100% removal of phenol was achieved by extending the operation days. The results show that phenol degrading bacteria are able to revitalize their metabolic conditions for phenol degradation. The ability of the microorganisms to tolerate the salinity of the wastewater depends on the biomass adaptation to the carbon source.^48^ Since the biomass was fully acclimated to the carbon source, there was not a significant effect on the phenol removal efficiencies. Saline wastewater causes an increase in the membrane permeability of the bacteria leading to the poisoning effect of sodium on cells.^49^ Rotation of the media and the attached biofilm leads to the reduction of the permeability of the cell membrane; consequently, the presence of rotating biofilm in CASIR leads to the reduction of the degree of inhibition of salinity. However, salinity concentrations as high as 20 g L^−1^ contributed to the deterioration of the performance of the CASIR bioreactor, and it was considered the highest salinity level that could be tolerated by phenol degrading organisms. Phenol degradation in saline wastewater by SBR has also been reported by other researchers.^11,12,50^Fig. 6b illustrates the variations in DHA of the attached growth and suspended growth, where the DHA activity of the microbial content of the bioreactor is almost constant up to 15 g L^−1^ of salinity; further increase in salinity to 20 g L^−1^ led to the reduction of DHA. This is much more significant for the suspended biomass, indicating that attached growth biofilm is much more resistant to wastewater salinity.

### 3.6. Contribution of attached biofilm to overall performance of CASIR

Comparing the results of the DHA of suspended biomass and attached growth in the all operating conditions indicated that the DHA of the attached biofilm was higher than that of the suspended biomass. This is due to the higher resistance of the biofilm to the applied shocks.^51^ Higher DHA indicates that the biofilm had a significant impact on the overall performance of the CASIR. Higher activity of the biofilm may be related to the dynamic conditions in the inner layers of biofilm.^28^ In addition, higher local concentration of substrate and enzyme because of the presence of an inert carrier and polysaccharide matrix is the other reason for higher activity of biofilm.^28^ Furthermore, the effect of hydraulic shear and shear stress on the biomass could be the other reason for the higher activity of the biofilm. Rotation of the biofilm in the CASIR bioreactor increases the shear stress and also causes a thin layer of biofilm on the media and therefore, better contact between substrate and microorganism occurs.^52^ Consequently, the integration of rotating media with suspended solid in the CASIR bioreactor improves the overall performance of the bioreactor in the case of phenol removal in denitrifying conditions.

### 3.7. The effect of hydrogen peroxide injection

In order to biostimulate phenol degrading bacteria in the bioreactor, hydrogen peroxide was injected in a controlled flow rate by a syringe pump (Fig. 1). For this purpose, the bioreactor was operated at an inlet phenol concentration of 500 mg L^−1^, HRT of 12 h, media filing ratio of 40%, and MLSS of 4000 mg L^−1^. Table 3 shows the average steady-state conditions of bioreactor operation within 73 days. In the first phase, which was operated for 9 days, inlet nitrate concentration was 1190 mg L^−1^. The injection of hydrogen peroxide caused a slight reduction in the removal efficiencies of phenol and COD concentrations, compared with the conditions when nitrate was the only electron acceptor. Further reduction in removal efficiencies was observed when inlet nitrate concentration was reduced to 900 mg L^−1^. Under these conditions, the bioreactor deterioration in removal efficiency could be attributed to the toxicity of hydrogen peroxide to the microorganisms and their performance; because of this, toxicity was adversely affected.^38^ In addition, in the initial days of operation, the presence of nitrate in the inlet made the conditions favorable for denitrifying microorganisms to degrade phenol. After achieving the steady-state conditions, the H_2_O_2_/phenol ratio increased to 0.73 and inlet nitrate concentration was reduced to 600 mg L^−1^. The bioreactor was operated for 12 days under these conditions. As shown in the table, phenol and COD removal efficiencies became 59.9 and 33.7%, respectively. DHA of the biomass and biofilm also showed a considerable decrease, indicating that the presence of hydrogen peroxide resulted in the deterioration in the performance of the microorganisms. The results show that microbial activity ceased due to the presence of hydrogen peroxide. After reaching the steady-state conditions, the H_2_O_2_/phenol ratio was increased to 1.10 and nitrate concentration was reduced to 300 mg L^−1^. Operation of the bioreactor in these conditions led to an increase in phenol and COD removal efficiencies, showing that the microbial biomass was adapting to the presence of hydrogen peroxide. Measuring the DHA at the end of this operational run also showed that the activities of the microorganisms were increasing. PA also increased at the end of this operational run and it was measured to be 49 U g_biomass_^−1^ and 88 U g_biomass_^−1^ for attached growth and suspended growth, respectively. In the next step, inlet nitrate was completely removed and the H_2_O_2_/phenol ratio was kept constant at 1.10. After 7 days of operation in these conditions, complete degradation of phenol was reached and almost 80% of the COD was also removed. DHA of the biomass and biofilm was 9.8 and 14.68 μg TF per g_biomass_ per day, respectively. PA of the microorganisms also showed an increasing trend, confirming that the peroxidase enzyme was generated by microorganisms. In addition, the complete degradation of phenol showed that the microbial content of the bioreactor adapted to the presence of hydrogen peroxide and switching of the bioreactor from denitrifying conditions to peroxidase-mediated conditions was completed.

**Table 3:** Average steady-state phenol, COD and nitrate removal efficiencies as a function of H_2_O_2_/phenol ratio (inlet phenol concentration = 500 mg L^−1^, MLSS = 4000 mg L^−1^, HRT = 12 h, OLR = 1000 g m^−3^ d^−1^)

PA (attached)^b^	PA (suspended)^b^	DHA (attached)^b^	DHA (suspended)^a^	Nitrate RE ± SD (%)	COD RE ± SD (%)	Phenol RE ± SD (%)	Nitrate in	H_2_O_2_/phenol
7	15	6.01	2.1	50.49 ± 6.76	47.9 ± 1.47	58.4 ± 2.4	1190	0.36
8	14	5.1	1.5	57.66 ± 1.41	44.17 ± 1.9	56.9 ± 3.9	900	0.36
9	17	3.9	0.95	98.3 ± 1.51	33.7 ± 3.72	59.9 ± 8.45	600	0.73
88	49	9.7	5.7	99.86 ± 0.48	43.8 ± 10.74	78.16 ± 8.5	300	1.10
142	94	14.68	9.8	—	75.0 ± 6.12	98.3 ± 3.15	0	1.10
210	—	15.98	—	—	88.59 ± 0.75	100	0	0.73
221	—	16.25	—	—	86.87 ± 0.81	100	0	0.36
289	—	17.89	—	—	88.18 ± 0.78	100	0	0.55

a DHA is expressed in μg TF per g_biomass_ per day.

b PA is expressed in U g_biommass_^−1^.

After operation of the bioreactor for almost 50 days, it was observed that MLSS of the system was almost completely removed and all the suspended biomass was attached to the media. In the next experimental runs, the bioreactor was operated only with attached biofilm. This unpredicted change in the bioreactor could be due to the release of hydrophobic exopolymeric substances participating in the increment of cohesiveness of the suspended biomass.^53^ Chao *et al.*^54^ also reported that higher hydrophobicity was observed in biofilm compared to suspended activated sludge, suggesting that more hydrophobic bacteria existed in biofilms than in suspended activated sludge.

It is important to optimize the amount of injected hydrogen peroxide in the bioreactor for the enzymatic biodegradation of phenol. Therefore, the H_2_O_2_/phenol ratio was reduced to 0.73 at an inlet phenol concentration of 500 mg L^−1^. The bioreactor was operated for one week in these conditions. The results show that COD removal efficiency increased to 88.59%. DHA and PA were also showing an increasing trend, indicating that higher H_2_O_2_ concentration was toxic to microbial metabolism and leads in particular to the inactivation of the enzyme.^38^

In the next step, the H_2_O_2_/phenol ratio was reduced to 0.36. As shown in table, there was a slight reduction in the COD removal efficiency at this ratio, suggesting that injected H_2_O_2_ is not enough for the microbial biostimulation to generate the peroxidase enzyme. Therefore, this ratio was increased to 0.55 and it was observed that the COD removal efficiency increased to almost 88%. DHA and PA were also increased to 17.89 μg TF per g_biomass_ per day and 289 U g_biomass_^−1^, respectively; 0.55 was therefore considered the optimum value for the H_2_O_2_/phenol ratio.

**Fig. 7:**
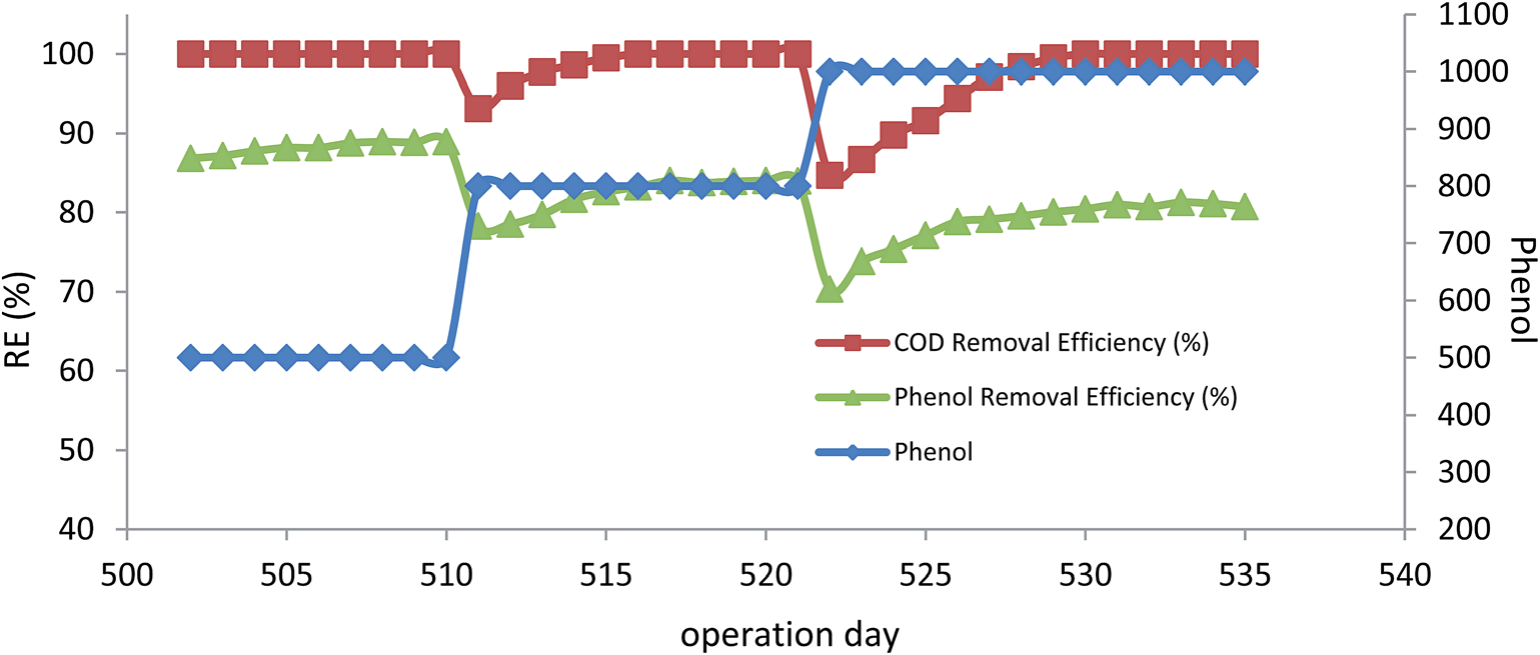
The effect of inlet phenol concentration in the peroxidase-mediated bioreactor. (HRT = 12 h, H_2_O_2_/phenol = 0.55).

After optimizing the H_2_O_2_/phenol ratio, the inlet phenol concentration increased to 800 mg L^−1^ and 1000 mg L^−1^ at the optimum H_2_O_2_/phenol ratio of 0.55 and HRT of 12 h. Fig. 7 illustrates the results of higher inlet phenol concentration in phenol and COD removal, where there was a slight reduction in removal efficiency of phenol after increasing the inlet concentration, but the bioreactor was able to re-attain its performance and the complete degradation of phenol was reached for both 800 and 1000 mg L^−1^. COD removal efficiency also showed the same trend, and almost 84% COD removal for 800 mg L^−1^ of inlet phenol and 80% for 1000 mg L^−1^ was reached. Measuring the microbial activity of the biofilm also showed that there was an increase in DHA. At the end of the experimental run for 500 mg L^−1^ and 1000 mg L^−1^ of phenol, DHA was 21.58 and 22.25 μg TF per g_biomass_ per day, respectively. The increase in the DHA indicates that the microbial activity was increased at higher concentrations and the acclimated biofilm was able to tolerate higher phenol loading rates. The PA of the biofilm remained almost unchanged and was found to be 295 and 290 U g_biomass_^−1^ for 800 and 1000 mg L^−1^ of the inlet phenol concentration, respectively. Microbial adaptation by H_2_O_2_ for total petroleum hydrocarbon also showed that PA at different experimental runs remained almost constant, ranging from 382–410 U g_biomass_^−1^.^38^

### 3.8. Comparison of the performance of the anoxic and enzymatic degradation of phenol

**Fig. 8:**
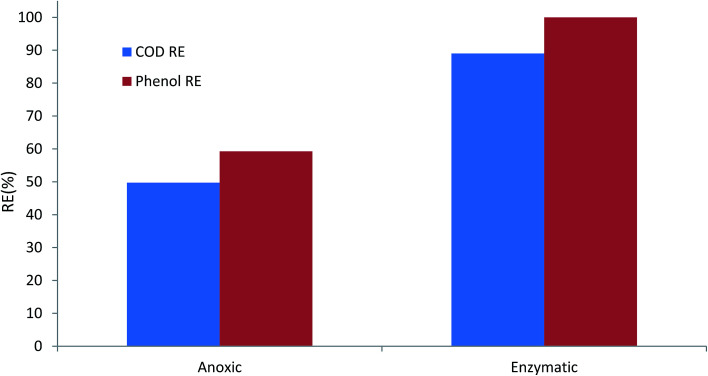
Comparison of the performance of anoxic and enzymatic degradation of phenol at the same loading rate of 1000 g m^−3^ d^−1^.

Fig. 8 illustrates the performance of anoxic and enzymatic bioreactors at the same phenol loading rate of 1000 g m^−3^ d^−1^. The phenol removal efficiency increased from almost 59% in anoxic conditions to 100% in enzymatic conditions. COD removal efficiency also increased considerably. Furthermore, the enzymatic bioreactor was able to tolerate phenol loading rates as high as 2000 g m^−3^ d^−1^, and complete degradation of phenol along with almost 81% COD removal efficiency was reached in the enzymatic process.

Comparing the DHA of the biofilm in anoxic and enzymatic processes also shows that microbial activity in the H_2_O_2_-induced process was also considerably higher, compared to anoxic conditions. The highest DHA for the H_2_O_2_-induced process was 22.25 μg TF per g_biomass_ per day, while it was 14.68 μg TF per g_biomass_ per day for anoxic conditions. Higher DHA indicates that the bacterial activity, and thus substrate consumption rate, increased. This could be due to the fact that the injection of H_2_O_2_ at a given concentration stimulated the bacteria, and therefore bacterial growth and biodegradation increased.^38^

Attaining such a high performance of the bioreactor for phenol degradation could be due to the presence of acclimated bacteria capable of phenol degradation. In addition, continuous exposure of the bacteria to low concentrations of hydrogen peroxide stimulated the bacteria to produce peroxidase enzyme and therefore phenol degradation took place.^55^

Comparing the results of peroxidase-mediated phenol degradation with anoxic conditions for phenol degradation revealed that biostimulation of bacteria with the injection of an inconsiderable amount of hydrogen peroxide into the CASIR bioreactor caused the *in situ* generation of large amounts of peroxidase enzyme, contributing to the degradation of phenol and associated COD.

## 4. Conclusion

The present study describes the performance of the CASIR bioreactor for the degradation of phenol in anoxic conditions and in the presence of hydrogen peroxide. The results reveal that the CASIR bioreactor could completely remove phenol and a large portion of the COD as well as nitrates in anoxic conditions. It was found that greater removal efficiencies were reached with a media filling ratio of 40%. Monitoring the DHA of the biofilm in the bioreactor revealed that the presence of biofilm in the bioreactor made the process more tolerant of applied shocks. It was also found that the CASIR bioreactor was able to completely remove the phenol loading rate of 667 g m^−3^ d^−1^ in anoxic conditions. The results show that the CASIR bioreactor is able to re-attain its performance after applying an organic loading shock. The salinity of the wastewater was also investigated and it did not affect the performance of the bioreactor. Switching the bioreactor operation from denitrifying conditions to peroxidase-mediated conditions improved the overall performance of the bioreactor. This was done due to the *in situ* generation of peroxidase enzyme as a result of microbial simulation by hydrogen peroxide. *In situ* generation of peroxidase enzyme in the bioreactor led to the complete degradation of phenol at the loading rate of 2000 g m^−3^ d^−1^. Finally, it can be concluded that using H_2_O_2_-induced microbial cells in a cyclic activated sludge is a promising technique for the enzymatic degradation of phenol and corresponding COD.

## Conflicts of interest

There are no conflicts to declare.

## Supplementary Material

## References

[cit1] Senthilvelan T. , Kanagaraj J., Panda R. C. and Mandal A. B., Clean Technol. Environ. Policy, 2014, vol. 16, pp. 113–126

[cit2] Kaczorek E., Smułek W., Zdarta A., Sawczuk A., Zgoła-Grześkowiak A. (2016). Ecotoxicol. Environ. Saf..

[cit3] EPA , Update of Human Health Ambient Water Quality Criteria: 2,4-Dimethylphenol 105-67-9, Office of Science and Technology Office of Water, EPA 820-R-15-085, 2015

[cit4] Li M., Wen D., Qiang Z., Kiwi J. (2017). RSC Adv..

[cit5] Zhang T., Cheng L., Ma L., Meng F., Arnold R. G., Sáez A. E. (2016). Chemosphere.

[cit6] Yang L., Wang B., Lai S., Jiang C., Zhong H. (2015). RSC Adv..

[cit7] Mekuto L., Ntwampe S. K. O., Akcil A. (2016). Sci. Total Environ..

[cit8] Garfí M., Flores L., Ferrer I. (2017). J. Cleaner Prod..

[cit9] Wu Y., Sun Q., Wang Y.-w., Deng C.-x., Yu C.-P. (2017). Ecotoxicol. Environ. Saf..

[cit10] Chen Y., He J., Wang Y.-Q., Kotsopoulos T. A., Kaparaju P., Zeng R. J. (2016). Biochem. Eng. J..

[cit11] Ferrer-Polonio E., García-Quijano N. T., Mendoza-Roca J. A., Iborra-Clar A., Pastor-Alcañiz L. (2016). Biochem. Eng. J..

[cit12] Ferrer-Polonio E., Mendoza-Roca J. A., Iborra-Clar A., Alonso-Molina J. L., Pastor-Alcañiz L. (2016). J. Ind. Eng. Chem..

[cit13] Liu Q., Singh V. P., Fu Z., Wang J., Hu L. (2017). Environ. Sci. Pollut. Res. Int..

[cit14] Grady C. P. L. , Daigger G. T., Love N. G. and Filipe C. D. M., Biological Wastewater Treatment, Third Edition, CRC Press, 2011

[cit15] Ramos A. F., Gomez M. A., Hontoria E., Gonzalez-Lopez J. (2007). J. Hazard. Mater..

[cit16] Ren L.-F., Chen R., Zhang X., Shao J., He Y. (2017). Bioresour. Technol..

[cit17] Muhamad M. H., Sheikh Abdullah S. R., Abu Hasan H., Abd Rahim R. A. (2015). J. Environ. Manage..

[cit18] Oberoi A. S., Philip L. (2017). J. Environ. Chem. Eng..

[cit19] Tziotzios G., Teliou M., Kaltsouni V., Lyberatos G., Vayenas D. V. (2005). Biochem. Eng. J..

[cit20] Yusoff N., Ong S.-A., Ho L.-N., Wong Y.-S., Mohd Saad F. N., Khalik W., Lee S.-L. (2016). Biochem. Eng. J..

[cit21] Leyva-Díaz J. C., Calderón K., Rodríguez F. A., González-López J., Hontoria E., Poyatos J. M. (2013). Biochem. Eng. J..

[cit22] Asif M. B., Nguyen L. N., Hai F. I., Price W. E., Nghiem L. D. (2017). Int. Biodeterior. Biodegrad..

[cit23] Alneyadi A. H., Ashraf S. S. (2016). Chem. Eng. J..

[cit24] Zappi M., White K., Hwang H. M., Bajpai R., Qasim M. (2000). J. Air Waste Manag. Assoc..

[cit25] Jafari S. J., Moussavi G., Yaghmaeian K. (2015). Bioresour. Technol..

[cit26] Moussavi G., Jafari S. J., Yaghmaeian K. (2015). Bioresour. Technol..

[cit27] Clesceri L. S. , Greenberg A. E. and Eaton A. D., Standard Methods for the Examination of Water and Wastewater, 20th Edition, APHA American Public Health Association, 1998

[cit28] Schneider I., Topalova Y. (2013). Biotechnol Biotechnol Equip..

[cit29] Xie J., Hu W., Pei H., Dun M., Qi F. (2008). Environ. Monit. Assess..

[cit30] Tandjaoui N., Tassist A., Abouseoud M., Couvert A., Amrane A. (2015). Biocatal. Agric. Biotechnol..

[cit31] El-Naas M. H., Al-Zuhair S., Makhlouf S. (2010). Chem. Eng. J..

[cit32] Sarfaraz S., Thomas S., Tewari U. K., Iyengar L. (2004). Water Res..

[cit33] Bajaj M., Gallert C., Winter J. (2010). Bioresour. Technol..

[cit34] Rosenkranz F., Cabrol L., Carballa M., Donoso-Bravo A., Cruz L., Ruiz-Filippi G., Chamy R., Lema J. M. (2013). Water Res..

[cit35] Chen T., Liu X., Zhang X., Chen X., Tao K., Hu X. (2016). Chemosphere.

[cit36] Byun I.-G., Nam H.-U., Song S. K., Hwang I.-S., Lee T.-H., Park T.-J. (2005). Korean J. Chem. Eng..

[cit37] Moussavi G., Ghorbanian M. (2015). Chem. Eng. J..

[cit38] Moussavi G., Shekoohiyan S., Naddafi K. (2017). Chem. Eng. J..

[cit39] Damayanti A., Ujang Z., Salim M. R., Olsson G. (2011). Water Sci. Technol..

[cit40] Li A.-j., Li X.-y., Yu H.-q. (2011). Process Biochem..

[cit41] Marañón E., Vázquez I., Rodríguez J., Castrillón L., Fernández Y., López H. (2008). Bioresour. Technol..

[cit42] Uygur A., Kargi F. (2004). Process Biochem..

[cit43] Moussavi G., Mahmoudi M., Barikbin B. (2009). Water Res..

[cit44] Juang R.-S., Kao H.-C., Tseng K.-J. (2010). Sep. Purif. Technol..

[cit45] Acikgoz E., Ozcan B. (2016). Int. Biodeterior. Biodegrad..

[cit46] Tyagi R. D., Du Y. G., Bhamidimarri R. (1996). Water Res..

[cit47] Li L., Suwanate S., Visvanathan C. (2017). Bioresour. Technol..

[cit48] Bassin J. P., Kleerebezem R., Muyzer G., Rosado A. S., van Loosdrecht M. C., Dezotti M. (2012). Appl. Microbiol. Biotechnol..

[cit49] Zhao W., Wang Y., Liu S., Pan M., Yang J., Chen S. (2013). Chem. Eng. J..

[cit50] Moussavi G., Barikbin B., Mahmoudi M. (2010). Chem. Eng. J..

[cit51] Daigger G. T. , Upgrading Wastewater Treatment Plants, Second Edition, Taylor & Francis, 1998

[cit52] Liu Y., Tay J. H. (2001). J. Appl. Microbiol..

[cit53] Jorand F., Boué-Bigne F., Block J. C., Urbain V. (1998). Water Sci. Technol..

[cit54] Chao Y., Guo F., Fang H. H. P., Zhang T. (2014). Colloids Surf., B.

[cit55] Yanto D. H. Y., Tachibana S. (2014). Chemosphere.

